# The importance of safe lithium plasma monitoring in older people

**DOI:** 10.1192/bjb.2025.10163

**Published:** 2026-06

**Authors:** Judy S. Rubinsztein, Steven Willis, Harleen Birgi, Kapila Sachdev, Christopher Southwell, Andrew P. Stewart, John T. O’Brien

**Affiliations:** 1 Department of Psychiatry, University of Cambridge, Cambridge, UK; 2 Cambridgeshire and Peterborough Foundation Trust, Fulbourn Hospital, Cambridge, UK; 3 Norfolk and Suffolk NHS Foundation Trust, Bury, UK; 4 East London NHS Foundation Trust, London, UK; 5 Craigavon Area Hospital, Portadown, Northern Ireland; 6 Cambridge University Hospitals NHS Foundation Trust, Cambridge, UK

**Keywords:** Lithium, old age psychiatry, bipolar type I or II disorders, renal failure, recurrent unipolar depression

## Abstract

We consider that the current National Institute for Health and Care Excellence (NICE) guideline CG185 on bipolar disorder does not provide sufficiently specific guidance for the safe monitoring of plasma lithium levels in older people. We feel this needs correction, and laboratories across the UK should lower the range recommended for monitoring older people’s lithium levels in line with guidelines from the International Society for Bipolar Disorder. This would provide a safety net in older people in order to prevent lithium toxicity without compromising efficacy.

Lithium is acknowledged as the gold standard for maintenance treatment in bipolar disorder, effective in mania and moderately so in bipolar depression.^1^ It is also used to augment antidepressants in treatment-resistant depression, is effective and safe for prophylaxis in recurrent unipolar depression^2,3^ and may offer neuroprotective benefits.^4^ Lithium has been shown to reduce the risk of suicide and psychiatric admissions in both recurrent unipolar and bipolar disorders.^3^ Shulman and colleagues^4^ discuss that 50% of people with bipolar disorder will be over 60 years old by 2030.^3^ This demographic shift underscores the need for safe lithium use, with initial monitoring when starting, followed by regular 3-monthly monitoring of lithium plasma levels 12 h post-dose in the first year, then 6 monthly thereafter, as well as monitoring of renal function, including estimated glomerular filtration rate (eGFR), thyroid function and calcium levels, at least every 6 months.^1^

## Lithium levels and older adults

Older patients will usually require lower doses of lithium to achieve therapeutic blood levels, owing to age-related pharmacokinetic and pharmacodynamic changes, polypharmacy, drug–drug interactions and comorbidities, which may increase the risk of lithium toxicity.^5^ The current reference range for lithium in UK laboratories is 0.4–1.0 mmol/L, with some variations globally.^4^ National Institute for Health and Care Excellence (NICE) guidelines recommend maintaining plasma lithium levels between 0.6 and 0.8 mmol/L for first-time users and between 0.8 and 1.0 mmol/L for those who relapse or have subthreshold symptoms.^1^ However, these guidelines do not specify lithium level monitoring in an age-specific way. Lithium toxicity, which often manifests as neurotoxicity (87%), nephrotoxicity (47%) and cardiovascular toxicity (45%), can become a serious concern, especially in older patients, many of whom receive polypharmacy.^5^ Sun et al, in their systematic review of case reports of lithium toxicity in older people, suggest that significant adverse effects or lithium toxicity may occur at levels of over 0.8 mmol/L in over-75-year-olds.^5^

## Adverse effects: the renal system

Since the renal system almost entirely clears lithium, reduced renal function, which is common in older people, increases the risk of lithium accumulation, necessitating lower oral doses. Lithium-induced nephrogenic diabetes insipidus can cause polyuria, secondary thirst and sometimes irreversible damage. Although the condition may be reversible within the first 2–6 years, about 20% of patients develop irreversible diabetes insipidus. Lithium has also been implicated in renal damage seen in pathological samples, such as cortical scarring and microcysts, collectively referred to as lithium-induced nephropathy.^6^

A systematic review and meta-analysis found that about 25% of lithium-treated patients developed chronic kidney disease (CKD), a significantly higher prevalence than in the general population (at around 5%).^6^ In patients aged 65 and older, this prevalence increased to one-third of individuals. The risk of CKD is more pronounced with prolonged lithium use, with a two-fold higher risk compared with non-lithium treatments. However, factors such as body mass index, smoking, hypertension and diabetes did not significantly influence CKD risk, although cardiovascular disease showed a near-significant association.^6^ Although there is some controversy regarding the extent of CKD risk associated with lithium versus other treatments,^7^ older people appear to be at a higher risk. Individuals with higher levels of lithium (above 0.8 or 1.0 mmol/L) were at higher risk of acute kidney injury.^7^ Rej et al demonstrated in a population cohort study that lithium levels above 0.7 mmol/L increase the risk of renal decline, whereas levels below or equal to 0.7 mmol/L do not lead to this risk in older populations.^8^

## Safe and effective dosing

The question of whether, even at lower levels, lithium is safe (from a renal perspective) in older people without affective disorder has been addressed to some extent. Aprahamian and colleagues examined renal safety in older people receiving the drug for mild cognitive impairment in a placebo-controlled trial. In this study, there were no adverse renal effects with lithium use at low doses (150–600 mg daily) over 2 years.^9^ The target lithium range in this study was 0.25–0.5 mmol/L, lower than what is typically used for bipolar disorder.

There is also evidence from a cohort study^2^ and a randomised controlled trial^10^ in people with unipolar or bipolar disorder that dosage reduction (by 25–50%) (lithium levels between 0.45 and 0.79 mmol/L)^10^ significantly reduced affective morbidity. In addition, thyroid-stimulating hormone levels were decreased in this group, as were total side-effects and tremor.^10^

Two Delphi studies have addressed the issue of whether lower levels of lithium meet the right balance of being both safe and effective in older adults with bipolar disorder. An international group of experts for the International Society for Bipolar Disorder (ISBD) reached consensus on using plasma monitoring of 0.4–0.8 mmol/L for those aged 60–79 and 0.4–0.7 mmol/L for those over 80.^4^ A second Delphi study of old age psychiatrists in Germany showed that there was consensus on slightly lower plasma levels, of 0.4–0.7 mmol/L for patients under 80 and 0.4–0.6 mmol/L for those aged 80 and over.^11^ Experts in both studies agree that maintaining lower lithium concentrations is effective and safer for older patients, given concerns about kidney function and neurotoxicity. A group of us in the UK, from the Faculty of Old Age Psychiatry of the Royal College of Psychiatrists, prepared a talk on this topic for the Faculty’s annual conference held in Liverpool in 2025, presenting this evidence base. We surveyed the audience of predominantly old age psychiatrists, trainees and staff grades (93 in in total), who voted after the talk. The highest consensus was on the question asking whether we should recommend change to lithium monitoring for people over 65 years old in laboratories in the UK and 82% agreed, 9% disagreed and 9% were not sure. In a further question about whether they agreed with the ISBD guidance, 84% agreed, 3% disagreed and 8% were unsure. So, the largely UK audience supported by a large majority that current lithium plasma monitoring advice needs to be changed.

## Implications for clinical practice

In the UK, it is becoming common practice for old age psychiatrists to aim for lower lithium levels. However, this approach may not be known among general practitioners, who would naturally follow local laboratory advice and national NICE guidelines, which do not specify that lower plasma doses should be used in older people. Current British National Formulary (BNF) guidance (2025) states that lithium blood tests should be done weekly until concentrations are stable, then 3 monthly for the first year and then 6 monthly.^12^ The ISBD’s guidelines (2019), based on their Delphi survey, differ slightly in this regard and more pragmatically recommend 3–6 monthly monitoring of lithium.^4^

If renal impairment develops, nephrology consultation is recommended, and the pros and cons of continuing lithium treatment must be weighed carefully.

On the basis of the somewhat limited literature on the topic, the results of published Delphi expert consensus studies and our own survey reported here, we consider that more conservative and safer lithium monitoring practices should be adopted in the UK for older people, in line with current clinical opinion, such as those suggested by the ISBD.^4^ Interestingly, in line with our views, the BNF recently changed its advice (in March 2025) and now suggests that initial serum lithium concentrations should be between 0.4 and 0.6 mmol/L for patients aged 65 years and older, adjusted according to response on specialist advice. This lower range is also specifically recommended in the prophylaxis of recurrent unipolar depression (for all ages).^12^ We accept that sometimes slightly higher levels may be needed in older people during a manic episode, so prefer the advice from the ISBD, but agree with these new initial levels recommended by the BNF.

## Conclusion

Lithium remains a very important treatment for bipolar disorder and treatment-resistant unipolar depression, including in older people, but its use in older individuals requires careful consideration of dose adjustments, frequent monitoring and potential alternatives, owing to the risk of nephrotoxicity and other age-related concerns. We call for implementation strategies to encourage laboratories to change their reference values for lithium levels for older adults in line with the ISBD guidance (2019).^4^

## About the authors

**Judy S. Rubinsztein**, MBChB, FRCPsych, PhD (Cantab), PGCert MedEd, is a consultant in old age psychiatry at Fulbourn Hospital, Cambridge, an Affiliated Assistant Professor in the Department of Psychiatry at the University of Cambridge, and an Executive Member of the Faculty of Old Age Psychiatry, Royal College of Psychiatrists, London, UK. **Steven Willis**, MBBS LLM, MRCPsych, is a consultant psychiatrist and Associate Medical Director at Bury North Older Peoples Community Mental Health Team, Norfolk and Suffolk NHS Foundation Trust, Bury, UK. **Harleen Birgi**, MBBS, MRCPsych, PGCME, FHEA, is a Specialty Trainee with Tower Hamlets Community Mental Health Team, East London NHS Foundation Trust, London, and a Higher Trainee Representative on the Faculty of Old Age Psychiatry Executive Committee, Royal College of Psychiatrists, London, UK. **Kapila Sachdev**, MBBS, MRCPsych, is a consultant old age psychiatrist with Tower Hamlets Mental Health Care of Older People Community Team, East London NHS Foundation Trust, London, and Regional Representative to the Faculty of Old Age Psychiatry, Royal College of Psychiatrists, London, UK. **Christopher Southwell**, MB BCh BAO, MRCPsych, is a consultant in old age psychiatry and Clinical Director of Psychiatry of Old Age and Memory Services, Southern Trust, in the Bluestone Unit, Craigavon Area Hospital, Portadown, Northern Ireland. **Andrew P. Stewart**, MB, BChir MA (Cantab), PhD, is a consultant in nephrology and general internal medicine with Cambridge University Hospitals, based in Addenbrooke’s Hospital, Cambridge, UK. **John T. O’Brien**, DM, FMedSci, is Professor of Old Age Psychiatry in the Department of Psychiatry, University of Cambridge School of Clinical Medicine, Cambridge, and an honorary consultant old age psychiatrist with Cambridgeshire and Peterborough NHS Foundation Trust, Cambridge, UK.
